# Clinical Bureau of Am. Inst. of Homœopathy

**Published:** 1882-01

**Authors:** T. F. Pomeroy

**Affiliations:** Chn. Bur. Clin. Med. Am. Inst. Hom.


### CLINICAL BUREAU OF AM. INS. OF HOMŒOPATHY.

The following circular has been sent to each member of the Bureau of Clinical Medicine. It is confidently expected that a prompt response to the same will be forwarded to the chairman that the report may be both full and complete.

The Bureau is constituted as follows, viz:

David Thayer, M. D., Boston; N. F. Cooke, M. D., Chicago; J. C. Morgan, M. D., Philadelphia; P. G. Valentine, M. D., St. Louis; S. Lilienthal, M. D., New York; Wm. M. Cote, M. D., Washington; Edward Rushmore, M. D., Plainfield. N. J.; John W. Dowling. M. D., New York; Adolph Lippe, M. D,, Philadelphia; T. S. Mitchell, M. D., Chicago; N. R. Morse, M. D., Salem, Mass.; E. A. Farrington, M. D., Philadelphia; Albert R. Barrett, M. D., Richmond, Va.; T. F. Pomeroy, M. D., Jersey City, N. J., *Chairman.*

547 *Bramhall Avenue, Jersey City, Nov.* 1, 1881.

MY DEAR DOCTOR:

Section 1 of Art. vii of our By-Laws provides that a Bureau of Clinical Medicine shall be appointed annually whose duty it is to report on "Diagnosis and General and Special Therapeutics." The papers constituting the report of this Bureau are thus restricted to those subjects, and cannot include others that are assigned to other bureaus of the Institute; and, as under our law of cure and system of practice, diagnosis relates to remedies as well as to pathological states, the symptomatic phenomena both of disease and drugs in their relations to each other constitute our therapeutics. The results therefore of our observation and experience in the adaptation of drug symptoms, objective and subjective, to those of disease, comprehend the legitimate sphere of action of this bureau as its title plainly indicates.

In the selection of its members I have sought to embrace the largest field of observation possible in the use of proved drugs of our materia medica; both in their single and concurrent use, and in all attenuations (with the reasons for the selection both of the drug and of the attenuation), and the results of their action, that our report may be both comprehensive and complete.

You will please, therefore, select the subject of your paper accordingly, and at the earliest possible date report the same to me, to the end that all such adjustments of subjects as may be requisite may be made without unnecessary delay, and in order to prevent confusion and needless repetition.

Fraternally and cordially yours,

T. F. Pomeroy, M. D.,

*Chn. Bur. Clin. Med. Am. Inst. Hom.*

